# Cost-effectiveness of modified fully oral 9-month treatment regimens for rifampicin-resistant tuberculosis in Belarus, Georgia, Kazakhstan and the Republic of Moldova

**DOI:** 10.1136/bmjgh-2024-018099

**Published:** 2025-11-08

**Authors:** Kasim Allel, Tom Palmer, Gerard Joseph Abou Jaoude, Oleksandr Korotych, Askar Yedilbayev, Valentina Vilc, Andrei Corloteanu, Mariana Macari, Cula Evghenia, Dumitru Laticevschi, Ismailov Shakhimurat-Shaimovich, Rakisheva Anar-Saduakasovna, Tulepova Gulzhan-Elbrusovna, Daria Anatolievna-Ryazanet, Aimbekova Shahrizada-Yergalymovna, Natalia Yatskevich, Alena Skrahina, Dmitry Zhurkin, Zaza Avaliani, Nana Kiria, Nino Lomtadze, Nino Kiria, Teona Avaliani, Irma Khonelidze, Maka Danelia, Corina Maxim, Hassan Haghparast-Bidgoli, Jolene Skordis

**Affiliations:** 1 Nuffield Department of Population Health, University of Oxford, Oxford, Oxfordshire, UK; 2 Institute for Global Health, University College London, London, UK; 3 World Health Organization Regional Office for Europe, Copenhagen, Denmark; 4 Institute of Phthisiopneumology, Chisinau, Moldova (the Republic of); 5 The Global Fund to Fight AIDS Tuberculosis and Malaria, Grand-Saconnex, Genève, Switzerland; 6 National Scientific Center of Phthisiopulmonology of the Republic of Kazakhstan, Almaty, Kazakhstan; 7 Republican Scientific and Practical Center for Pulmonology and Tuberculosis, Minsk, Belarus; 8 National Center for Tuberculosis and Lung, Tbilisi, Georgia; 9 National Center for Disease Control and Public Health, Tbilisi, Georgia

**Keywords:** Tuberculosis, Health economics

## Abstract

**Introduction:**

Prior to 2020, treatment options for multidrug-resistant tuberculosis (MDR-TB) were limited and typically involved long treatment durations and high financial burdens. In the eastern European and central Asian (EECA) region, traditional inpatient tuberculosis (TB) care models, alongside high MDR-TB rates, escalate nosocomial transmission risks and treatment costs. Modified, fully oral, shorter treatment regimens (mSTR) implemented in the WHO European Region under operational research conditions offered a potential reduction in the burden of MDR-TB treatment for both patients and health systems.

**Methods:**

We conducted the first regional evaluation of the cost-effectiveness of the novel mSTR treatment regimen compared with the standard of care (SOC) in Belarus, Georgia, Kazakhstan and Republic of Moldova. We used cohort data on mSTR efficacy and WHO data on SOC in patients with MDR-TB. We used a Markov model, with treatment costs calculated from the provider perspective. Outcomes were measured in quality-adjusted life years (QALYs), with incremental cost-effectiveness ratios (ICER) calculated per QALY gained in each country. An annual 3% discount rate was used for both costs and outcomes. We performed univariate and probabilistic sensitivity analysis (PSA) to assess the robustness of our cost-effectiveness calculations under varying assumptions. Finally, we estimated potential cost savings if mSTR was implemented nationally and we evaluated the incremental net monetary benefit (iNMB) and willingness-to-pay (WTP) thresholds based on Wood *et al*’s country-level cost-effectiveness thresholds. All costs were reported in 2022 USD.

**Results:**

We estimated that mSTR can reduce TB treatment costs by between 23% and 47% and drug costs by 39% to 74%, compared with SOC in the countries studied. mSTR resulted in cost savings of between $3596 and $8174 per patient and offered additional health gains of between 0.56 to 2.69 QALYs per patient. mSTR remained cost-effective (iNMB>0) compared with SOC in 78%, 85%, 91% and 92% of PSA simulations in Belarus, Georgia, Kazakhstan and Republic of Moldova, respectively, when compared with their country-level WTP threshold. Implementing mSTR in up to 80% of MDR/rifampicin-resistant TB patients may result in cost savings of $20.5, 2.5, 0.7 and 0.2 million in Kazakhstan, Belarus, Republic of Moldova and Georgia; equivalent to 17%, 3%, 4% and 1% of their national TB budgets, respectively.

**Conclusions:**

Compared with SOC, mSTR is a more cost-effective treatment option for MDR/RR-TB, which should be considered by policymakers in the EECA region. Using insights from current implementations to scale up, plan operational changes and reallocate savings from mSTR could greatly enhance TB services and patient care.

**WHAT IS ALREADY KNOWN ON THIS TOPIC**

- There is limited evidence for short tuberculosis (TB) regimens where the eligibility for most updated 6 months multidrug-resistant/rifampicin-resistant tuberculosis (MDR/RR-TB) treatments are restricted, such as in the eastern European and central Asian (EECA) area.

**WHAT THIS STUDY ADDS**

- We present a cost-effectiveness analysis of a modified 9 month fully oral treatment regimen (mSTR) for MDR/RR-TB across four EECA countries. We found significant cost savings and health benefits, establishing mSTR as a financially and therapeutically superior option and marking this as the first comprehensive study of its kind on mSTR.

**HOW THIS STUDY MIGHT AFFECT RESEARCH, PRACTICE OR POLICY**

- Recent findings highlight an 83% success rate and favourable safety profile for mSTR, supporting its adoption. Additionally, potential economic savings could enhance TB programmes, improving service access, care quality and reducing patient costs, aiding national and global TB control efforts.

## Introduction

 In 2022, 10.6 million people developed tuberculosis (TB), and 410 000 people fell ill with rifampicin-resistant (RR) or multidrug-resistant (MDR) TB globally,^1^ causing substantial mortality and morbidity.^2^,^4^ Although Europe accounts for only 2.2% of the global TB burden, it accounts for 16.3% of all MDR/rifampicin-resistant (RR) TB cases.^5^ Approximately 80% of those MDR/RR-TB cases are in the eastern Europe and central Asia (EECA) region. Treatment options for isoniazid or RR TB are limited and generally involve long treatment durations.^6 7^ In many EECA countries, TB treatment has traditionally prioritised hospitalisation, which escalates costs, for both patient and health system and increases the risk of nosocomial TB transmission. This approach is compounded by additional challenges such as fragmented health systems, high levels of migration and incarceration, and significant HIV prevalence.^8^,^10^ These risk factors underpin the pressing need for new, shorter and cheaper alternative antibiotic treatment for TB that minimises time in hospital and on treatment.^9 11 12^

In 2020, the WHO recommended the use of a 9 month oral standard short treatment regimen (STR) for TB.^13^ However, its adoption in the EECA region was limited due to widespread resistance to the components of this regimen.^5^ In response to this challenge, WHO’s European Office initiated operational research across 13 countries.^14^ This research aimed to assist these nations in transitioning to an all-oral 9 month regimen, where the standard STR components that faced resistance were substituted with new or repurposed drugs such as linezolid, cycloserine or delamanid. In line with literature supporting effectiveness and cost-effectiveness,^15 16^ new WHO guidelines in 2022^17^ prioritised simplified 6- or 9 month oral bedaquiline, pretomanid and linezolid with and without moxifloxacin BPaL(M) or clofazimine (BPaLC) regimens, which can contribute to further reduced costs and treatment burden for MDR/RR-TB patients and healthcare systems.^7^ However, pretomanid, a key component of BPaL(M), is not accessible in all countries,^18^ leading to limited programme adoption and the persistent use of longer treatment alternatives in the EECA region.^19^ Accessibility to pretomanid is often driven by high prices, regulatory delays, restricted procurement pathways and omission from national treatment guidelines regionally.

Consequently, to meet local needs and endorse alternative short oral regimens, WHO Europe and the Global Fund initiated the adoption of an alternative modified, fully oral, shorter treatment regimen (mSTR), boosting care quality, patient retention and treatment success rates for MDR/RR-TB.^14^ A single-arm cohort study conducted under operational research conditions in the EECA region found overall treatment success rates of 83% for mSTR.^20 21^ Here, we use effectiveness estimates from this cohort study to conduct the first comprehensive cost-effectiveness analysis of mSTR, compared with the current standard of care (SOC), for MDR/RR-TB patients across four EECA countries and detail both overall and per-patient economic costs and savings.

## Methods

We evaluated the cost-effectiveness of mSTR for people diagnosed with MDR/RR-TB, when compared with SOC, in Belarus, Georgia, Kazakhstan and Republic of Moldova. Our study followed the Consolidated Health Economic Evaluation Reporting Standards 2022 (CHEERS 2022) checklist for quality reporting economic evaluations^22^ and adhered to ISPOR (The Professional Society for Health Economics and Outcomes Research) modelling good practices.^23^

### Intervention and comparator treatment regimens

We evaluated mSTR as implemented through operational research conducted by the WHO in each country.^14 24^ This regimen had a treatment duration of 39 weeks and consisted of levofloxacin, bedaquiline, linezolid, clofazimine and cycloserine. For patients with suspected resistance or intolerance of cycloserine, delamanid was used instead. Initial dosing of drugs followed WHO 2019 recommendations and subsequently changed to WHO 2022 recommendations once available.^17 25^ The intervention was compared with the current provision of relevant long-SOC regimens (online supplemental text 1). If short-term SOC treatment was provided, long-term and short-term SOC treatments were standardised and converted into one measurement for comparability. The ‘SOC’ comparator can therefore be considered as representing the average cost and outcomes of all non-mSTR regimens in use at the time of data collection.

### Model description

We developed a Markov model to represent progression of a cohort of patients through multiple health states following treatment with either mSTR or SOC regimens. Individuals were assumed to enter the model with active RR- or MDR-TB, at the age of 40 years based on average age of notified MDR/RR-TB cases reported by WHO.^26^ Patients could transition through health states monthly, including treatment completion, loss to follow-up (LTFU), serious adverse events, treatment failure and death. The model structure is presented in online supplemental figure 1 with its respective associated text. Patients in the LTFU health state could reinitiate treatment each month. On failure of mSTR or SOC regimens, individuals were assigned to an alternative second-line (SL) or rescue treatment, equivalent to an individualised longer treatment regimen. Patients who had completed treatment could relapse following completion at a monthly rate of 2.8%^27^ for both treatments. Patients experiencing relapse also received the SL or rescue treatment. Following treatment completion, patients were assumed to enter the cured health state after a period of 12 months. People with unresolved TB or LTFU were assumed to have a probability of death of 6.86% per month.^28^ We considered a monthly likelihood of return to care after LTFU of 2.3%.^29^ Patient outcomes and economic costs were totalled over a 20 year time horizon, with all parameters and transitions modelled on a monthly cycle. Model parameters are reported in table 1.

**Table 1:** Model parameters, by country and treatment arm

Variable	Belarus	Georgia	Kazakhstan	Moldova	Source
Gross domestic product per capita (2021) in USD	7302	5023	10 379	5231	WB^59^
People diagnosed with MDR/RR-TB in 2021 (notifications, number of cases)	801	187	3755	593	WHO^3^
mSTR MDR/RR-TB treatment outcomes probabilities, based on country-specific treatment duration*					
Successful treatment (%)†	90.69	82.83	93.92	89.32	NTP^14^
Failed or unresolved (%)	2.61	1.01	3.38	3.88	NTP^14^
Treated but died (%)	3.91	1.01	2.03	0.97	NTP^14^
Lost to follow-up (%)	2.79	15.15	0.68	5.83	NTP^14^
Adverse effects (%)‡	19.63	1.87	4.12	9.91	NTP^14^
Resolved (%)§	64.71	50.00	62.50	61.54	NTP^14^
Resolved with sequelae (%)§	0.74	0.00	0.00	15.38	NTP^14^
Not resolved (%)§	2.94	0.00	0.00	0.00	NTP^14^
Dead (%)	31.62	50.00	37.50	23.08	NTP^14^
Conventional or standard MDR/RR-TB treatment outcomes probabilities (SOC), based on country-specific treatment duration*					
Successful treatment (%)†	82.90	78.44	76.14	69.23	WHO^3^
Failed and unresolved (%)	1.68	1.83	3.33	6.26	WHO^3^
Treated but died (%)	8.29	5.96	10.18	13.60	WHO^3^
Lost to follow-up (%)	7.12	13.76	10.35	10.91	WHO^3^
Adverse effects (%)‡	23.51				Lan *et al*^60^
Resolved (%)	69.70				Lan *et al*^60^
Resolved with sequelae (%)	0.00				Lan *et al*^60^
Not resolved (%)	19.50				Lan *et al*^60^
Dead (%)	10.80				Lan *et al*^60^
Second-line MDR/RR-TB treatment outcomes probabilities (based on XDR-TB, annually)					
Successful treatment (%)	71.78	56.60	72.67	56.76	WHO^3^
Failed and unresolved (%)	6.62	9.43	8.40	18.92	WHO^3^
Treated but died (%)	8.36	7.55	12.52	10.81	WHO^3^
Lost to follow-up (%)	13.24	26.42	3.36	13.51	WHO^3^
Utility weights					
MDR-TB patients with no cure	0.68 (‘SE’=0.14)				Jit *et al*^30 31^
TB patients with treatment completed or cured	0.81 (SE=0.04)				Jit *et al*^30 31^
Patients lost to follow-up or end of life care	0.68 (SE=0.14)				Jit *et al*^30 31^
Adverse effects	0.68 (SE=0.14)				Jit *et al*^30 31^
Death	0.00				N/A
Costs (2022 USD)					
Average cost per patient for mSTR	7681	7161	5759	6311	NTP^14^
Treatment duration for mSTR (in days)	273	273	272	273	NTP^14^
Average cost per patient for comparator	14 532	10 832	13 168	8240	NTP^14^
Treatment duration for comparator (in days)	467	505	600	547	NTP^14^

* Country-specific treatment durations are detailed at the end of this table under ‘Costs’. Probabilities for each treatment arm depended upon treatment duration and were consecutively transformed to show monthly changes: all parameters and transitions were modelled on a monthly cycle.

† Successful treatment is defined as those patients with cured and completed treatments; we grouped them because categories were equal among SOC treatments due to missing detailed information.

‡ Percentage of patients with at least 1 severe adverse event.

§ NTP data were adapted, and these proportions were calculated utilising the number of severe events reported because data were not reported per patient but in number of severe adverse events. For instance, the proportion of individuals recovered is equal to those recovered from severe adverse events divided by all severe adverse events observed. Typically, patients did not exhibit more than one severe adverse event.

MDR, multidrug resistance; N/A, not applicable; NTP, National Tuberculosis Control Programme; RR, rifampicin-resistant; SOC, standard of care; TB, tuberculosis; XDR, extensively drug-resistant.

### Treatment outcomes and effectiveness

We considered treatment success or completed, failed or unresolved, LTFU, cured and treated but died. Treatment outcomes for the mSTR intervention were extracted from the WHO mSTR operational research database for each country.^14^ As data were unavailable for specific comparator regimens, treatment outcomes for SOC regimens were based on outcomes for the 2019 MDR/RR-TB cohort in each country, extracted from the WHO Global TB database.^3^ In the absence of data, treatment outcomes for the SL or rescue regimen following treatment failure were assumed to be the same as outcomes for extensively drug-resistant (XDR) TB as reported in the WHO Global TB database. These outcomes were used to calculate monthly transition probabilities based on regimen-specific treatment durations. Quality-adjusted life years (QALYs) gained were calculated as an outcome of the cost-effectiveness analysis. Utility weights used for QALYs were extracted from the literature^30 31^ based on the EuroQol five-dimension (EQ-5D) instrument (table 1). QALYs were discounted at a rate of 3% annually in the base case analysis.^32^

### Economic costs

**Figure 1:**
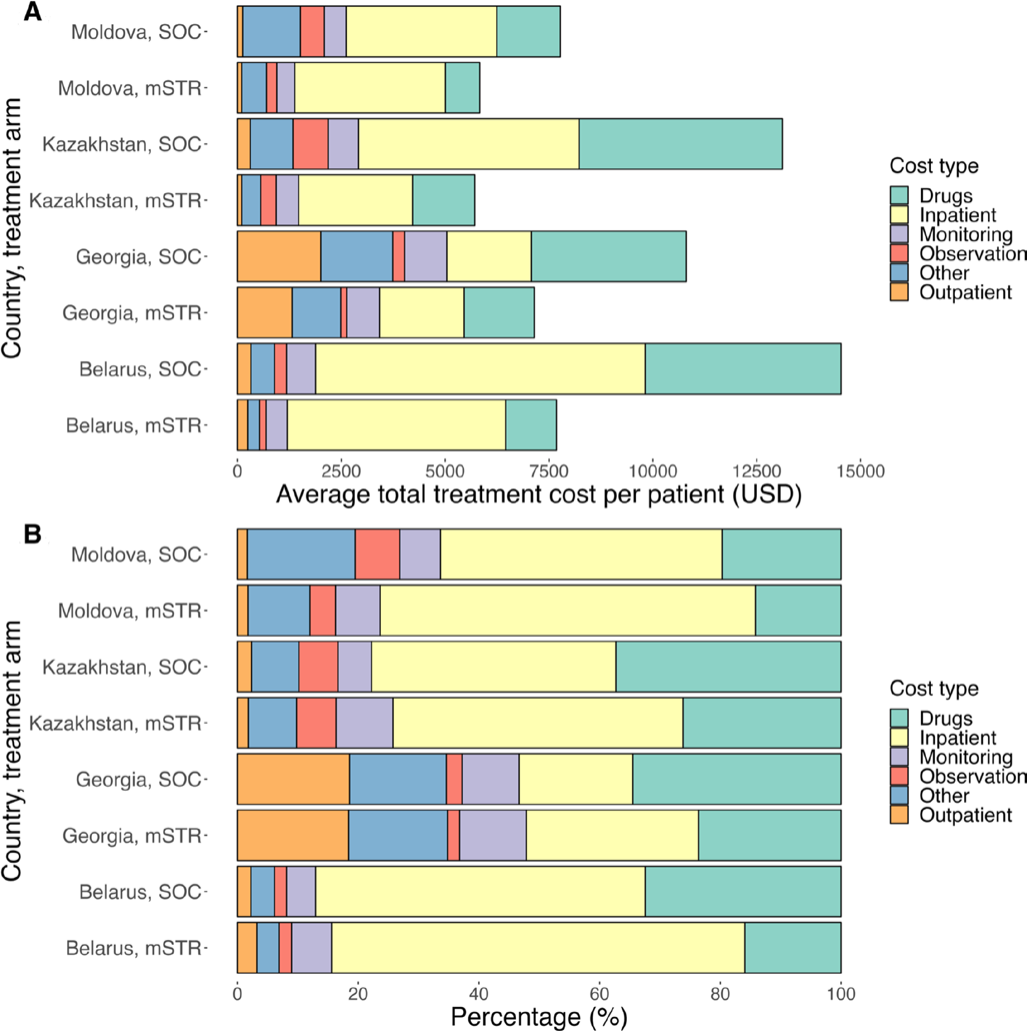
Treatment regimen costs (excluding adverse events treatment costs). ‘Other’ comprised average costs for psychosocial support, home-based care and programme management. mSTR, modified shorter all-oral treatment regime; SOC, standard of care; USD, US dollars.

Country-specific unit costs were estimated from a provider perspective using ingredients-based (bottom-up) cost analysis of primary data, provided by the National Tuberculosis Programme for each of the four EECA countries. Countries were asked to report average costs for all patients receiving short/long SOC regimens (Text A1). Costs were collated in a customised Excel data collection tool and are summarised in figure 1. Data collection tools for each country are provided in onlinesupplemental files A1A4. SL or rescue treatment costs were assumed to be equal to SOC costs, which may underestimate costs of treatment following failure. Due to a lack of data, the cost of treating serious adverse events was assumed equal to the cost of 1 month of TB treatment, excluding drug costs. Patients who were LTFU were assumed to receive a monthly phone call, at an estimated cost of US$1 per month in each country.^33^ All costs were expressed in 2022 USD, converted using 2022 exchange rates specific to each country, and discounted at 3% in the base case analysis.^32^ All drugs in our regimens are available via the Global Drug Facility, but EECA countries typically lack the necessary provisions to procure through it. Cost descriptions and country-specific exchange rates used are presented in online supplemental tables 1 and 2.

### Uncertainty and sensitivity analyses

We performed three sensitivity analyses. First, we conducted a univariate sensitivity analysis on key cost and outcome components, by employing a ±25% change in value for each parameter to assess their impacts on ICERs. We evaluated mSTR and conventional treatment costs, SL or rescue treatment costs, return-to-care after LTFU rate, discount rate (0%, 3% and 6%) and utility weights for cured/treated and untreated patients. Time horizon and relapse rates were analysed, but no significant difference was found in costs or outcomes.

Second, we conducted a probabilistic sensitivity analysis (PSA) using 1000 Monte Carlo simulations to simultaneously model variation in parameters based on assumed distributions. A Beta distribution was assumed for all probabilities and utility weights, while a gamma distribution was assumed for economic costs. Related probabilistic outcomes were modelled by sampling a single probability and deriving its complement to ensure they summed to one. As most 95% CI for parameters were absent, we approximated a scaling factor for the shape parameter for gamma distributions based on observed means/percentages in sensitivity analyses. Following standardised health evaluation methods, we estimated the Cost-Effectiveness Acceptability Curve (CEAC) or percentage of total simulations that were cost-effective following a willingness-to-pay (WTP) per QALY gained equal to country-level cost-effectiveness thresholds, previously estimated by Woods and colleagues.^34^

We also calculated incremental net monetary benefit (iNMB) across PSA simulations using a range of WTP thresholds, as suggested in recent literature.^35^ We calculated the percentage of positive iNMB from all PSA simulations using country WTPs. Subsequently, we estimated the Expected Value of Perfect Information (EVPI)^36^ to assess the value of resolving decision uncertainty. EVPI was defined as the difference between the expected net benefit under perfect information and the maximum expected net benefit given current uncertainty.

Third, to examine the potential impact of operational research conditions for mSTR on our results, we conducted a conservative scenario analysis where treatment outcomes assumed the same for mSTR and SOC regimens, using WHO’s reported treatment outcomes for the 2019 MDR/RR-TB treatment cohort in each country.^3^ mSTR treatment was delivered under operational research conditions, which should broadly reflect programme conditions. However, there remains some uncertainty in likely implementation fidelity and treatment outcomes if implemented outside of these conditions. While the same medical team served both the mSTR and SOC groups and there was no observed difference in adherence support, medical staff may have been more vigilant in monitoring adverse effects from mSTR since reporting was pivotal and outcome data are more complete for the mSTR cohort. Conversely, higher numbers of individuals who were not evaluated in the SOC cohort may lead to overestimated success rates (ie, data may be more likely to be missing for those with negative treatment outcomes).

### Costs at scale

We calculated total costs at scale per treatment arm using our estimated costs per patient and WHO’s reported notification for MDR/RR-TB individuals per country.^3^ Costs at scale were reported as total costs under different scenarios assuming varied coverage levels of mSTR between current coverage up to a maximum of 100% of MDR/RR-TB cases. Treatment costs were also compared as a proportion of the total national TB budget in US$, which includes domestic, Global Fund and grant-related income.^37^ We reported countries’ cost-savings from scale-up of mSTR in full and via exploring three scenarios: 0% mSTR coverage (ie,everyone receives SOC), current mSTR coverage in each country, and 80% mSTR coverage (ie,an arbitrary assumed upper limit for mSTR scale-up to account for eligibility concerns). The proportion of the total TB budget in each country that is accounted for by MDR/RR-TB treatment was considered.

All analyses were conducted in R and all relevant code is publicly available on GitHub (https://bit.ly/CEmSTR_EECAregion).

### Patient and public involvement

Patients and the public were not directly involved in the design or reporting of the study.

## Results

### Analysis of unit costs

Average mSTR treatment costs per patient were $7682, $7161, $5711 and $6251, compared with SOC costs of $14 533, $10 831, $13 121 and $8179 in Belarus, Georgia, Kazakhstan and Republic of Moldova, respectively (figure 1 and online supplemental table 3). In all countries except Georgia, inpatient care for mSTR was the largest cost component. Belarus and Kazakhstan saw lower inpatient costs for mSTR due to shorter hospital stays, while in Georgia and the Republic of Moldova, inpatient costs were the same for mSTR and SOC. Drug costs were lower for mSTR in all countries, with reductions of 74% in Belarus, 54% in Georgia, 69% in Kazakhstan and 39% in Moldova vs SOC.

### Base-case cost-effectiveness analysis results

The mSTR intervention was less costly and more effective in all countries and deemed to dominate SOC (table 2). Across the four countries, mSTR is associated with average cost savings of between $3596 and $8174 per patient, and health gains of 0.56–2.69 QALYs per patient. Dynamics over time per health-state group and country are shown in online supplemental figures 2–5.

**Table 2:** Base case cost-effectiveness analysis results, by country and treatment arm

Country, treatment	QALYs per person	Total cost per person ($)	Incremental QALYs gained per person	Incremental costs per person ($)	Incremental cost-effectiveness ratio ($/QALY)
Belarus					
SOC	9.28	19 970			
mSTR	9.84	12 812	0.56	−7157	Dominant
Georgia					
SOC	8.64	14 543			
mSTR	9.73	9572	1.09	−4972	Dominant
Kazakhstan					
SOC	c9.09	17 613			
mSTR	c10.99	9439	1.90	−8174	Dominant
Moldova					
SOC	8.11	10 902			
mSTR	10.80	7305	2.69	−3596	Dominant

mSTR, modified shorter all-oral treatment regimen; QALY, quality-adjusted life years; SOC, standard of care.

### Sensitivity analyses

In all countries, mSTR remained the dominant strategy in all univariate sensitivity analyses (table 3; Online supplemental figures 6 and 7). Incremental costs were most sensitive to changes in SL or rescue treatment costs following failure in Belarus and Kazakhstan, reflecting comparatively high SOC costs in these two countries. In Georgia and the Republic of Moldova, incremental costs were more sensitive to changes in non-drug costs for SOC, reflecting comparatively low SOC drug costs. Incremental QALYs were most influenced by the discount rate, with utility weights playing a lesser role.

**Table 3:** Base case cost-effectiveness analysis results, by country

Sensitivity analyses	Country, change in value*	Incremental QALYs gained per person	Incremental costs per person ($)
One-way SA	**Belarus**		
Impacts on utilities			
**I.** Utility MDR RR-TB, unresolved, AE and LTFU	+25%	0.55	−7157
	−25%	0.57	−7157
**II.** Utility MDR RR-TB, cured	+25%	0.58	−7157
	−25%	0.47	−7157
Impacts on costs			
**IV.** Non-drug costs mSTR treatment	+25%	0.56	−5589
	−25%	0.56	−8727
**V.** Non-drug costs SOC treatment	+25%	0.56	−4684
	−25%	0.56	−9631
**VI.** Costs SL or rescue treatment	+25%	0.56	−7143
	−25%	0.56	−7172
Impacts on costs and utilities			
**VII.** Discount rate	0.00	0.80	−7684
	0.06	0.40	−6690
PSA	†	0.26	−9238
One-way SA	**Georgia**		
Impacts on utilities			
**I.** Utility MDR RR-TB, unresolved, AE and LTFU	+25%	1.08	−4972
	−25%	1.11	−4972
**II.** Utility MDR RR-TB, cured	+25%	1.13	−4972
	−25%	0.95	−4972
Impacts on costs			
**IV.** Non-drug costs mSTR treatment	+25%	1.09	−3463
	−25%	1.09	−6480
**V.** Non-drug costs SOC treatment	+25%	1.09	−3142
	−25%	1.09	−6801
**VI.** Costs SL or rescue treatment	+25%	1.09	−5274
	−25%	1.09	−4669
Impacts on costs and utilities			
**VII.** Discount rate	0.00	1.55	−5557
	0.06	0.79	−4554
** **PSA	†	0.83	−6479
One-way SA	**Kazakhstan**		
Impacts on utilities			
**I.** Utility MDR RR-TB, unresolved, AE and LTFU	+25%	1.88	−8174
	−25%	1.93	−8174
**II.** Utility MDR RR-TB, cured	+25%	1.97	−8174
	−25%	1.66	−8174
Impacts on costs			
**IV.** Non-drug costs mSTR treatment	+25%	1.90	−7138
	−25%	1.90	−9209
**V.** Non-drug costs SOC treatment	+25%	1.90	−10 187
	−25%	1.90	−6160
**VI.** Costs SL or rescue treatment	+25%	1.90	−7964
	−25%	1.90	−8383
Impacts on costs and utilities			
**VII.** Discount rate	0.00	2.64	−8919
	0.06	1.41	−7528
PSA	†	1.51	−11 328
One-way SA	**Moldova**		
Impacts on utilities			
**I.** Utility MDR RR-TB, unresolved, AE and LTFU	+25%	2.66	−3596
	−25%	2.72	−3596
**II.** Utility MDR RR-TB, cured	+25%	2.82	−3596
	−25%	2.30	−3596
Impacts on costs			
**IV.** Non-drug costs mSTR treatment	+25%	2.69	−2298
	−25%	2.69	−4895
**V.** Non-drug costs SOC treatment	+25%	2.69	−5185
	−25%	2.69	−2008
**VI.** Costs SL or rescue treatment	+25%	2.69	−4043
	−25%	2.69	−3149
Impacts on costs and utilities			
**VII.** Discount rate	0.00	3.76	−4078
	0.06	1.97	−3180
PSA	†	1.82	−4181

* Change in values was expressed as ±25% variations for each parameter, except for discount rates, to evaluate impacts on incremental QALYs and ICER.

† For PSA ‘probability sensitivity analyses’, we used a gamma distribution for costs, and Dirichlet distribution for utilities and probabilities; all parameters were varied at once following their respective distributions. Univariate sensitivity analyses for each country are graphically depicted in online supplemental figures 6 and 7. PSA is graphically depicted by treatment arm (mSTR and SOC) and country in online supplemental figures 8–11.

AE, adverse events; LTFU, loss to follow-up; MDR-TB, multidrug-resistant TB; mSTR, modified shorter all-oral treatment regimen; PSA, probability sensitivity analyses; QALY, quality-adjusted life years; RR-TB, rifampicin-resistant TB; SL, second line; SOC, standard of care.

**Figure 2:**
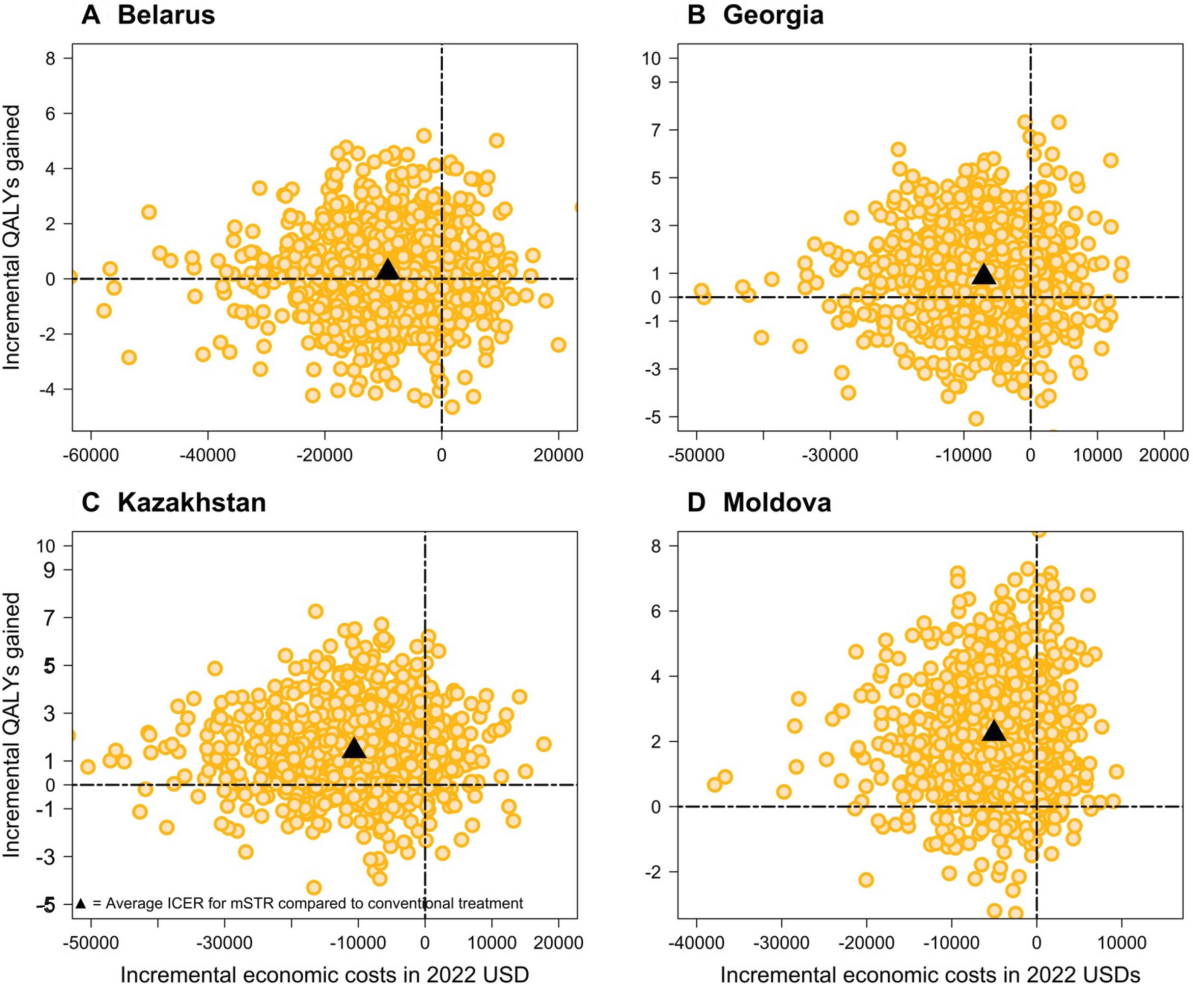
Estimation of the probabilistic sensitivity analysis for costs and QALYs, by country. ICER, incremental cost-effectiveness ratio; mSTR, modified short treatment regimen; QALYs, quality-adjusted life years; USD, US dollars.

The mSTR intervention remained cost-effective in most PSA simulations (table 3, figure 2). In Kazakhstan and the Republic of Moldova, 85% and 84% of PSA simulations indicated increased QALYs and reduced costs (online supplemental figures 8–11). This figure was lower for Belarus and Georgia, 62% and 56% respectively (online supplemental figures 12–15), given relatively high SOC success rates and rates of adverse events. Online supplemental figure 16 displays traditional CEACs for mSTR, compared with SOC.

The estimated mean iNMB from Monte Carlo PSA simulations was $10 714 (IQR: 1085–19 752) in Belarus, $8973 (2423–13 897) in Georgia, $24 951 (11 205–37 769) in Kazakhstan and $7261 (4022–11 860) in Moldova (online supplemental figure 17). The proportion of PSA simulations yielding a positive iNMB was 78%, 85%, 91% and 92% respectively. The EVPI per patient was approximately $1780, $501, $872 and $206 (online supplemental figure 18), respectively, indicating comparatively high residual decision uncertainty for Belarus.

In scenario analyses, mSTR remained cost-saving in every country, even if assuming equal outcomes for mSTR and SOC (online supplemental table 4). Incremental QALYs were slightly negative in Belarus and Georgia, reflecting differences in observed adverse events, but mSTR remained cost-saving despite having the lowest SOC duration among sampled countries.

### Costs at scale

Current total expenditure on MDR/RR-TB treatment was estimated at $9.8, $1.6, $47.7 and $4.7 million, representing 13.2%, 13.9%, 39.9% and 28.0% of the national TB budget in Belarus, Georgia, Kazakhstan and Republic of Moldova, respectively (online supplemental figure 19). Scaling up mSTR to a maximum of 80% of MDR/RR-TB patients in each country is associated with cost savings relative to current coverage levels of $2.5 (3% of total national TB budget), $0.2 (1%), $20.5 (17%) and $0.7 (4%) million in each country, respectively (online supplemental figure 20). Scaling up mSTR in Kazakhstan offers significant cost savings due to the high volume of MDR/RR-TB notifications and the currently low mSTR coverage.

## Discussion

We found that the mSTR regimen for MDR/RR-TB was more effective and less costly than SOC comparator regimens in Belarus, Georgia, Kazakhstan and Republic of Moldova, generating cost-savings and additional health gains as alternative treatment. In light of our findings, policymakers in Belarus, Georgia, Kazakhstan and the Republic of Moldova, and the EECA region more widely, should consider implementing mSTR regimens at a national scale, specifically in settings where pretomanid is not yet available.

Prior studies have assessed the cost-effectiveness of short course BPaL-based regimens for RR-TB. Two studies assessed the cost-effectiveness and financial impact of the BPaL regimen for pre-XDR TB and treatment-resistant MDR-TB patients, using data from the Nix trial in six countries from Africa and Asia.^38 39^ In Indonesia, Kyrgyzstan and Nigeria, BPaL implementation was projected to cut XDR-TB related treatment costs by 15%–32%.^39^ In South Africa, Georgia and the Philippines, BPaL proved to be cost-effective, with outcomes hinging on drug pricing and follow-up adherence.^38^ A recent study, using data from the TB PRACTECAL trial, found that the BPaL 6 month, all-oral treatment was cost-saving compared with SOC in India, Georgia, Philippines and South Africa, saving $112–$1173 per person.^15^ A subsequent study reported similar savings in Belarus and Uzbekistan.^40^

In contrast, Rosu *et al* determined that a 9 month all-oral bedaquiline-containing regimen was cost-ineffective in Ethiopia, India, Moldova and Uganda, due to significantly high drug costs (around 15%–25% of total provider costs).^41^ They found an ICER=$5966 per QALY gained, not deemed cost-effective at a WTP threshold of $2400.^41^ However, the control regimen in this study excluded bedaquiline, reducing drug costs, while more recent WHO guidelines recommend including bedaquiline in both short- and long-course regimens. Our analysis confirms that short-course, all-oral regimens for RR-TB are likely to yield cost savings and broaden the evidence base to encompass a mSTR-based treatment regimen. Given that the mSTR regimen can be used without age restrictions, during pregnancy and breastfeeding, and in settings without pretomanid access, our analysis expands on the existing evidence base of economic evaluations of BPaL-based regimens.

While shortening treatment duration is one aspect of increasing treatment acceptability and patient-centred care, long initial hospitalisation periods also impact patients negatively and remain a substantial cost driver in the EECA region.^42^ For example, in Belarus, patients were treated in hospital for 90 days under the mSTR regimen and 150 days under SOC. Reduced hospitalisation can reduce provider and patient costs,^43^ and nosocomial transmission.^8^ However, transitioning away from hospital-focused care in some EECA countries may require complex changes to care models, including clinical guidelines and funding mechanisms.^10^

The scale-up of short-course regimens can also have a substantial influence on overall TB expenditure, which can help ensure continued progress towards national and international TB control targets.^44^ Although we could not employ a societal perspective, implementing mSTR could significantly cut TB-related costs for patients and their families, crucial for the 87% of global MDR/RR-TB patients facing catastrophic expenses.^3^

The inclusion of bedaquiline has significantly enhanced treatment outcomes and lowered mortality rates for drug-resistant TB,^45^ with all long-course regimens in Belarus, Georgia, Kazakhstan and the Republic of Moldova incorporating it by 2022. While this study does not specify regimen components due to the emerging risk of bedaquiline resistance,^46^,^49^ careful regimen selection is vital. Addressing bedaquiline resistance^50^,^53^ necessitates further research on companion drugs and adjunct therapies,^54^ alongside clinical trials.^26 55^ Moreover, strategies like meticulous treatment monitoring, ensuring continuous drug supply and advancing rapid bedaquiline susceptibility testing are critical to combating drug-resistant TB.^56 57^ Although shorter regimens might reduce resistance by fostering better treatment adherence, these considerations are essential for future TB programme planning and economic assessments.

The study has some limitations. Primary data were not available for certain key parameters, including rates of relapse and adverse events. Additionally, outcomes data for the comparator relies on 2019 cohort data from the WHO TB database. Although this helps to ensure comparability across countries, outcomes are not associated with specific regimens, and it offers a pragmatic estimate of possible comparator outcomes. The impact of the mSTR intervention on transmission, and thereby reinfection, was also not included in this analysis, nor was the possible impact on bedaquiline resistance. Microbiological endpoints for relapse and acquired bedaquiline resistance were not available but should be the subject of future research. Cost comparisons between countries should be interpreted with caution, especially for Georgia which uses a diagnosis-related group payment model, meaning that hospitals are reimbursed a fixed amount per TB case regardless of treatment regimen or length of stay. Limited data were available to inform the health-related quality of life of patients experiencing adverse events. Also, the uptake of BPaL or other short treatments, that are neither the mSTR nor included under the SOC, may impact cost-effectiveness potentially affecting SOC costs. However, at the time of analysis, pretomanid, a component of BPaL, was not widely accessible in the EECA region. Finally, data on the number of fluoroquinolone-resistant TB cases and associated costs were not available. Fluoroquinolone resistance may increase health and economic burden,^58^ and therefore SOC estimates may not be fully generalisable to the fluoroquinolone-sensitive population which mSTR was tested in. Despite these potential limitations, our study is the first economic evaluation of the mSTR regimen and uses the largest multinational cohort study in the EECA region, providing cost and health outcome estimates with treatment provision close to programme conditions.

The mSTR regimen for MDR/RR-TB is likely to be effective and cost saving when compared with current SOC in Belarus, Georgia, Kazakhstan and the Republic of Moldova. We found further significant cost savings at scale if adherence to mSTR treatment is improved, especially among countries with the lowest mSTR coverage (eg., Kazakhstan and Republic of Moldova). Savings from implementing mSTR may be retained by national TB programmes and used to further improve TB outcomes and progress toward WHO’s End TB strategy goal of 90% treatment success rate for TB towards 2030.

## Supplementary material

10.1136/bmjgh-2024-018099 online supplemental file 1

10.1136/bmjgh-2024-018099 online supplemental file 2

10.1136/bmjgh-2024-018099 online supplemental file 3

10.1136/bmjgh-2024-018099 online supplemental file 4

10.1136/bmjgh-2024-018099 online supplemental file 5

## Data Availability

All data relevant to the study are included in the article or uploaded as supplementary information.
