# Supplementary material for: Cost-effectiveness of modified fully oral 9-month treatment regimens for rifampicin-resistant tuberculosis in Belarus, Georgia, Kazakhstan and the Republic of Moldova
Source: BMJ Glob Health. 2025 Nov 8;10(11):e018099. doi: 10.1136/bmjgh-2024-018099 (PMC12603705; doi:10.1136/bmjgh-2024-018099)
Supplement: online supplemental file 1 [file bmjgh-10-11-s001.docx]

#### Supplementary appendix

Cost-effectiveness of modified fully oral nine-month treatment regimens for rifampicin-resistant tuberculosis in Belarus, Georgia, Kazakhstan and Republic of Moldova

Table of Contents

[Supplemental Text 1. Long SOC treatment specifications	3](#_Toc197078401)

[Supplemental Table 1. Provider cost components for mSTR and standard MDR/RR-TB care	5](#_Toc197078402)

[Supplemental Table 2. USD and country-specific exchange rates used	6](#_Toc197078403)

[Supplemental Table 3. Treatment costs excluding adverse events in 2022 USD	6](#_Toc197078404)

[Supplemental Table 4. Outcome scenario cost-effectiveness results	7](#_Toc197078405)

[Supplemental Table 5. GDP per capita Cost-effectiveness thresholds used as willingness-to-pay values, by country	7](#_Toc197078406)

[Supplemental Table 6. CHEERS 2023 Checklist	7](#_Toc197078407)

[Supplemental Figure 1. Markov model diagram	11](#_Toc197078408)

[Supplemental Figure 2. Health states dynamics overtime by treatment arm, Belarus	13](#_Toc197078409)

[Supplemental Figure 3. Health states dynamics overtime by treatment arm, Georgia	14](#_Toc197078410)

[Supplemental Figure 4. Health states dynamics overtime by treatment arm, Kazakhstan	15](#_Toc197078411)

[Supplemental Figure 5. Health states dynamics overtime by treatment arm, Moldova	16](#_Toc197078412)

[Supplemental Figure 6. Univariate sensitivity analyses impact on incremental QALYs, by country	17](#_Toc197078413)

[Supplemental Figure 7. Univariate sensitivity analyses impact on incremental economic costs, by country	18](#_Toc197078414)

[Supplemental Figure 8. Probability sensitivity analysis impacts on economic costs and QALYs per treatment arm, Kazakhstan	19](#_Toc197078415)

[Supplemental Figure 9. Percentage of simulations per incremental ICER and QALY scenarios, Kazakhstan	20](#_Toc197078416)

[Supplemental Figure 10. Probability sensitivity analysis impacts on economic costs and QALYs per treatment arm, Moldova	21](#_Toc197078417)

[Supplemental Figure 11. Percentage of simulations per incremental ICER and QALY scenarios, Moldova	22](#_Toc197078418)

[Supplemental Figure 12. Probability sensitivity analysis impacts on economic costs and QALYs per treatment arm, Belarus	23](#_Toc197078419)

[Supplemental Figure 13. Percentage of simulations per incremental ICER and QALY scenarios, Belarus	24](#_Toc197078420)

[Supplemental Figure 14. Probability sensitivity analysis impacts on economic costs and QALYs per treatment arm, Georgia	25](#_Toc197078421)

[Supplemental Figure 15. Percentage of simulations per incremental ICER and QALY scenarios, Georgia	26](#_Toc197078422)

[Supplemental Figure 16. Costs-effectiveness acceptability curves (CEAC), by country	27](#_Toc197078423)

[Supplemental Figure 17. Incremental Net Monetary Benefit (iNMB) across WTP thresholds	28](#_Toc197078424)

[Supplemental Figure 18. Expected Value of Perfect Information (EVPI) across different WTP thresholds, by country	29](#_Toc197078425)

[Supplemental Figure 19. Costs and cost-savings at scale	30](#_Toc197078426)

[Supplemental Figure 20. Impact of mSTR scale-up on MDR/RR-TB treatment expenditure, by country	31](#_Toc197078427)

[References	33](#_Toc197078428)

Excel files per country and treatment arm with cost ingredient description and analyses are provided separately in Supplementary Excel file A.

# Supplemental Text 1. Long SOC treatment specifications

Standard of care (SOC) for MDR/RR TB consists of drug treatment for 18-20 months comprising drugs categorised into three groups (A, B, and C)^1^. Group A consists of three priority drugs recommended for inclusion in all regimens, whenever possible. These drugs are levofloxacin/moxifloxacin, bedaquiline, and linezolid. Group B comprises two additional drugs that may be considered for inclusion in all regimens, although it is not mandatory. These drugs are clofazimine and cycloserine/terizidone. Group C encompasses "other" agents, including injectables, which can serve as substitutes when it becomes necessary to complete a regimen with a minimum of four drugs, and agents from groups A and B cannot be utilised.

A full list of exact treatment drugs following SOC treatment by country is detailed below.

**Georgia**

| **Drug costs by regimen** | **Regimen cost (full cost 2022), US $** | **Number initiated** |
| --- | --- | --- |
| **MDR/RR-TB treatment (long-course comparator regimen)** |  |  |
| Lfx + Bdq + Lzd + Cfz + Cs | 3000 | 38 |
| Bdq + Lzd + Cfz + Cs+Dlm | 7000 | 7 |
| 8Am-Meropenem(Am)-Bdq(Dlm)-Lzd-Cfz-Cs-PAS-(E/Z)/10 Bdq(Dlm)-Lzd-Cfz-Cs-PAS-(E/Z) | 8000 | 7 |
| **MDR/RR-TB treatment (short course comparator regimen)** | | |
| Bdq+Pa+Lzd | 1200 | 12 |

**Kazakhstan**

| **Drug costs by regimen** | **Regimen cost (full cost 2022), US $** | **Number initiated** |
| --- | --- | --- |
| **MDR/RR-TB treatment (long-course comparator regimen)** | | |
| Bdq + Lzd + Cfz + Cs+Dlm+Z | 4897 | 458 |

**Moldova**

| **Drug costs by regimen** | **Regimen cost (full cost 2022), US $** | **Number initiated** |
| --- | --- | --- |
| **MDR/RR-TB treatment (long-course comparator regimen)** | | |
| 6Bdq+Cfz+Cs+Lfx+Lzd/12Cfz+Cs+Lfx+Lzd | 1612 | 839 |
| 6Bdq+Cfz+Cs+Lzd+Mfx/12Cfz+Cs+Lzd+Mfx | 1637 | 270 |

**Belarus**

| **Drug costs by regimen** | **Regimen cost (full cost 2022), US $** | **Number initiated** |
| --- | --- | --- |
| **MDR/RR-TB treatment (long-course comparator regimen)** | | |
| 6 Bdq Mfx Lzd Cfz Cs/ 14Mfx Lzd Cfz Cs | 2606 | 2 |
| 18 Bdq Lfx Lzd Cfz Cs | 3145 | 484 |
| 20 Bdq Mfx Lzd Cfz Cs | 3214 | 31 |
| 20 Bdq Lzd Cfz Cs Dlm | 8254 | 338 |
| 6-8 Bdq Lzd Cfz Cs Imp/Amx/Clv /  14-12 Bdq Lzd Cfz Cs | 5921 | 29 |
| 6-8 Bdq Lzd Cfz Cs Dlm Imp/Amx/Clv\ 14-12 Bdq Lzd Cfz Cs Dlm | 11588 | 12 |
| 20 Bdq Lzd Cfz Dlm | 8139 | 256 |
| **MDR/RR-TB treatment (short course comparator regimen)** | | |
| Bdq + Pa + Lzd +Mfx | 820 | 204 |
| Bdq + Pa + Lzd + Cfz | 929 | 138 |
| Bdq + Pa + Lzd | 778 | 9 |

Notes: Lfx refers to levofloxacin, Bdq to bedaquiline, Lzd to linezolid, Cfz to clofazimine, and Cs to cycloserine. Dlm stands for delamanid, Am for amikacin, and Meropenem is used as written. PAS denotes para-aminosalicylic acid, while E and Z refer to ethambutol and pyrazinamide, respectively. Mfx stands for moxifloxacin, Imp for imipenem, and Amx/Clv for the combination of amoxicillin and clavulanate. Pa refers to pretomanid.

# Supplemental Table 1. Provider cost components for mSTR and standard MDR/RR-TB care

| **Perspective** | **Cost component** | **Description** |
| --- | --- | --- |
| Provider | Baseline screening and ongoing monitoring during and after treatment | Cost of routine TB and non-TB related baseline tests and ongoing treatment/post-treatment monitoring |
|  | Treatment drug regimen | Cost of mSTR and standard MDR/RR-TB treatment regimens |
|  | Inpatient care | Average cost of an inpatient day for standard MDR/RR-TB treatment regimens and any mSTR patients where necessary |
|  | Outpatient care | Average cost of an outpatient visits for standard MDR/RR-TB treatment regimens and any mSTR patients where necessary |
|  | Adverse events | Cost of each relevant grade 3-4 adverse event, including inpatient care, outpatient care and drug costs |
|  | Other | Miscellaneous costs including care components such as psychosocial support |

Notes: mSTR= Modified short regimen. MDR= Multidrug resistance. RR= Rifampicin resistance. TB= Tuberculosis.

# Supplemental Table 2. USD and country-specific exchange rates used

| Country | Exchange rate | Source |
| --- | --- | --- |
| **Belarus** | 1 USD = 2.63 Belarusian Ruble | Value input by Belarus NTP |
| **Georgia** | 1 USD = 2.7 Georgian Lari | Value input by Georgia NTP |
| **Kazakhstan** | 1 USD = 469.42 Kazakhstani Tenge | Value input by Kazakhstan NTP |
| **Moldova** | 1 EUR= 19.90 Moldovan leu  1 USD = 0.95 EUR | Value input by Moldova NTP  Average exchange rate for 2022 (https://www.irs.gov/individuals/international-taxpayers/yearly-average-currency-exchange-rates) |

Notes: USD= United States Dollars; EUR=Euro. For Belarus, Georgia and Kazakhstan, data were reported in USD by the NTP, based on the above exchange rates. For Moldova, the NTP reported data in EUR based on the above exchange rate for Moldovan leu. This was then converted to USD for comparability with other countries.

# Supplemental Table 3. Treatment costs excluding adverse events in 2022 USD

| Country | Regimen | Drugs | Inpatient treatment costs | Outpatient treatment | Screening and monitoring costs | Treatment observation | Other | Total |
| --- | --- | --- | --- | --- | --- | --- | --- | --- |
| **Belarus** | mSTR | 1,225 | 5,259 | 248 | 509 | 161 | 280 | 7,682 |
| **Belarus** | SOC | 4,718 | 7,935 | 323 | 698 | 292 | 567 | 14,533 |
| **Georgia** | mSTR | 1,688 | 2,037 | 1,316 | 791 | 143 | 1,170 | 7,161 |
| **Georgia** | SOC | 3,729 | 2,037 | 2,006 | 1,019 | 282 | 1,733 | 10,831 |
| **Kazakhstan** | mSTR | 1,496 | 2,744 | 102 | 539 | 373 | 457 | 5,711 |
| **Kazakhstan** | SOC | 4,897 | 5,311 | 307 | 730 | 849 | 1,027 | 13,121 |
| **Moldova** | mSTR | 989 | 3,813 | 107 | 453 | 260 | 629 | 6,251 |
| **Moldova** | SOC | 1,618 | 3,813 | 133 | 553 | 604 | 1,458 | 8,179 |

# Supplemental Table 4. Outcome scenario cost-effectiveness results

| **Country, treatment** | **QALYs per person** | **Total cost per person**  **($)** | **Incremental QALYs gained per person** | **Incremental costs per person ($)** | **Incremental cost effectiveness ratio ($/QALY)** |
| --- | --- | --- | --- | --- | --- |
| **Belarus** |  |  |  |  |  |
| SOC | 9.30 | 20,950 |  |  | ICER: 54,083 |
| mSTR | 9.18 | 14,460 | -0.12 | -6,490 |  |
| **Georgia** |  |  | -0.18 | -3,554 | ICER: 19,744 |
| SOC | 8.64 | 14,543 |  |  |  |
| mSTR | 8.46 | 10,989 |  |  |  |
| **Kazakhstan** |  |  |  |  |  |
| SOC | 8.87 | 17,723 |  |  |  |
| mSTR | 9.09 | 10,800 | 0.22 | -6,923 | Dominant |
| **Moldova** |  |  |  |  |  |
| SOC | 8.13 | 11,237 |  |  |  |
| mSTR | 8.47 | 8,495 | 0.34 | -2,742 | Dominant |

Notes: SOC= Standard of care. mSTR= Modified shorter all-oral treatment regimen. QALY= Quality-adjusted life years.

# Supplemental Table 5. GDP per capita Cost-effectiveness thresholds used as willingness-to-pay values, by country

| Country | GDP per capita Cost-effectiveness threshold range in 2013 USDs* | GDP per capita Cost-effectiveness threshold range in 2022 USDs |
| --- | --- | --- |
| **Belarus** | 1,895 - 4,857 | 2,381– 6,102 |
| **Georgia** | 366 - 1,850 | 460 – 2,324 |
| **Kazakhstan** | 4,485 - 8,018 | 5,634 – 10,073 |
| **Moldova** | 148 - 1,151 | 186 - 1,446 |

Notes: *Cost effectiveness thresholds were extracted from Woods, Beth, et al. "Country-level cost-effectiveness thresholds: initial estimates and the need for further research." Value in Health 19.8 (2016): 929-935. GDP= Gross domestic product.

# Supplemental Table 6. CHEERS 2023 Checklist

| Topic | No. | Item | Location where item is reported |
| --- | --- | --- | --- |
| Title |  |  |  |
|  | 1 | Identify the study as an economic evaluation and specify the interventions being compared. | Title |
| Abstract |  |  |  |
|  | 2 | Provide a structured summary that highlights context, key methods, results, and alternative analyses. | Abstract |
| Introduction |  |  |  |
| Background and objectives | 3 | Give the context for the study, the study question, and its practical relevance for decision making in policy or practice. | Introduction section |
| Methods |  |  |  |
| Health economic analysis plan | 4 | Indicate whether a health economic analysis plan was developed and where available. | Methods section, second paragraph |
| Study population | 5 | Describe characteristics of the study population (such as age range, demographics, socioeconomic, or clinical characteristics). | Study population and settings section |
| Setting and location | 6 | Provide relevant contextual information that may influence findings. | Study population and settings section. |
| Comparators | 7 | Describe the interventions or strategies being compared and why chosen. | Interventions and comparators section |
| Perspective | 8 | State the perspective(s) adopted by the study and why chosen. | Costs section, first paragraph |
| Time horizon | 9 | State the time horizon for the study and why appropriate. | Methods section, second paragraph |
| Discount rate | 10 | Report the discount rate(s) and reason chosen. | Treatment outcomes section; costs section. |
| Selection of outcomes | 11 | Describe what outcomes were used as the measure(s) of benefit(s) and harm(s). | Treatment outcomes section |
| Measurement of outcomes | 12 | Describe how outcomes used to capture benefit(s) and harm(s) were measured. | Treatment outcomes section |
| Valuation of outcomes | 13 | Describe the population and methods used to measure and value outcomes. | Treatment outcomes section |
| Measurement and valuation of resources and costs | 14 | Describe how costs were valued. | Costs section |
| Currency, price date, and conversion | 15 | Report the dates of the estimated resource quantities and unit costs, plus the currency and year of conversion. | Costs section, first paragraph |
| Rationale and description of model | 16 | If modelling is used, describe in detail and why used. Report if the model is publicly available and where it can be accessed. | Model description section |
| Analytics and assumptions | 17 | Describe any methods for analysing or statistically transforming data, any extrapolation methods, and approaches for validating any model used. | Uncertainty and sensitivity analysis section |
| Characterising heterogeneity | 18 | Describe any methods used for estimating how the results of the study vary for subgroups. | Uncertainty and sensitivity analysis section |
| Characterising distributional effects | 19 | Describe how impacts are distributed across different individuals or adjustments made to reflect priority populations. | Uncertainty and sensitivity analysis section |
| Characterising uncertainty | 20 | Describe methods to characterise any sources of uncertainty in the analysis. | Uncertainty and sensitivity analysis section |
| Approach to engagement with patients and others affected by the study | 21 | Describe any approaches to engage patients or service recipients, the general public, communities, or stakeholders (such as clinicians or payers) in the design of the study. | Patient and public involvement section |
| Results |  |  |  |
| Study parameters | 22 | Report all analytic inputs (such as values, ranges, references) including uncertainty or distributional assumptions. | Methods section, |
| Summary of main results | 23 | Report the mean values for the main categories of costs and outcomes of interest and summarise them in the most appropriate overall measure. | Results section |
| Effect of uncertainty | 24 | Describe how uncertainty about analytic judgments, inputs, or projections affect findings. Report the effect of choice of discount rate and time horizon, if applicable. | Results section |
| Effect of engagement with patients and others affected by the study | 25 | Report on any difference patient/service recipient, general public, community, or stakeholder involvement made to the approach or findings of the study | Not applicable |
| Discussion |  |  |  |
| Study findings, limitations, generalisability, and current knowledge | 26 | Report key findings, limitations, ethical or equity considerations not captured, and how these could affect patients, policy, or practice. | Discussion section |
| Other relevant information |  |  |  |
| Source of funding | 27 | Describe how the study was funded and any role of the funder in the identification, design, conduct, and reporting of the analysis | Funding declaration |
| Conflicts of interest | 28 | Report authors conflicts of interest according to journal or International Committee of Medical Journal Editors requirements. | Competing interests declaration |

 From: Husereau D, Drummond M, Augustovski F, et al. Consolidated Health Economic Evaluation Reporting Standards 2022 (CHEERS 2022) Explanation and Elaboration: A Report of the ISPOR CHEERS II Good Practices Task Force. Value Health 2022;25. <doi:10.1016/j.jval.2021.10.008>^3^.

# Supplemental Figure 1. Markov model diagram

*Module structure and patient progression*

The Markov model identifies the care cascade for rifampicin-resistant or multidrug-resistant tuberculosis (RR/MDR-TB) patients starting from enrolment into the mSTR intervention arm or standard of care (SOC) comparator arm. All parameters are presented in the main manuscript Methods section and Table 1.

Patients flow through mutually exclusive states monthly using country-level transition probabilities and treatment durations. Upon initiation of treatment, patients transition into the "Still on treatment" state, where they may remain for several cycles, equivalent to the 9-month median mSTR duration (273 days) or longer for SOC regimens (467–600 days). All these probabilities were transformed to reflect monthly transitions between states accounting for treatment-specific durations and outcomes. Monthly transitions from SOC/mSTR treatment states can lead to completion of treatment, loss to follow-up (LTFU), side effects, failure of treatment, or death. LTFU patients have a likelihood of returning to care each month (2.3%), and unresolved or LTFU patients have a substantial monthly risk of death (6.86%).

Patients with severe adverse events become resolved, resolved with sequelae, not resolved, or dead, depending on outcome distributions. Patients who complete treatment enter temporary post-treatment status prior to curing (equivalent to one time cycle in the model), except where relapse has occurred. Relapse or failure of treatment leads to second-line (SL) or rescue treatment, depending on extensively drug-resistant TB (XDR-TB) treatment outcomes. SL treatment failure leads to transfer to the unresolved state.

Transition probabilities for each of the health states—successful treatment, mortality, LTFU, failure, and adverse event outcomes—were used from operational mSTR implementation data (NTP) and WHO sources. As illustrated, mSTR success rates ranged from 82.8% (Georgia) to 93.9% (Kazakhstan), LTFU from 0.68% (Kazakhstan) to 15.2% (Georgia), along with serious adverse events that occurred in up to 19.6% of mSTR patients. For SOC, success in treatment was lower and LTFU and death greater.

All states of health are linked to utility weights to estimate quality-adjusted life years (QALYs) between 0.68 (untreated disease or adverse events) and 0.81 (cure or completion of treatment). Death is assigned a utility of 0. Cost and outcomes were summed over the time horizon of 20 years. The model includes death in all states and models real-world care transitions with country-specific detail.

# Supplemental Figure 2. Health states dynamics overtime by treatment arm, Belarus

Notes: mSTR= modified shorter all-oral treatment regimen. MDR= multidrug, RR= Rifampicin-resistance. TB= tuberculosis.

# Supplemental Figure 3. Health states dynamics overtime by treatment arm, Georgia

Notes: mSTR= modified shorter all-oral treatment regimen. MDR= multidrug, RR= Rifampicin-resistance. TB= tuberculosis.

# Supplemental Figure 4. Health states dynamics overtime by treatment arm, Kazakhstan

Notes: mSTR= modified shorter all-oral treatment regimen. MDR= multidrug, RR= Rifampicin-resistance. TB= tuberculosis.

# Supplemental Figure 5. Health states dynamics overtime by treatment arm, Moldova

Notes: mSTR= modified shorter all-oral treatment regimen. MDR= multidrug, RR= Rifampicin-resistance. TB= tuberculosis.

# Supplemental Figure 6. Univariate sensitivity analyses impact on incremental QALYs, by country

Notes: Blue colours relate to increases in parameters while orange colours to decreases, following the impacts of +/-25% for each parameter on incremental QALYs.

# Supplemental Figure 7. Univariate sensitivity analyses impact on incremental economic costs, by country

Notes: Blue colours relate to increases in parameters while orange colours to decreases, following the impacts of +/-25% for each parameter on ICERs (incremental economic costs).

# Supplemental Figure 8. Probability sensitivity analysis impacts on economic costs and QALYs per treatment arm, Kazakhstan

Notes: QALYs= Quality-adjusted life years. mSTR= modified shorter all-oral treatment regimen. USD= United States dollars.

# Supplemental Figure 9. Percentage of simulations per incremental ICER and QALY scenarios, Kazakhstan

Notes: QALYs= Quality-adjusted life years. mSTR= modified shorter all-oral treatment regimen.

# Supplemental Figure 10. Probability sensitivity analysis impacts on economic costs and QALYs per treatment arm, Moldova

Notes: QALYs= Quality-adjusted life years. mSTR= modified shorter all-oral treatment regimen. USD= United States dollars.

# Supplemental Figure 11. Percentage of simulations per incremental ICER and QALY scenarios, Moldova

Notes: QALYs= Quality-adjusted life years. mSTR= modified shorter all-oral treatment regimen.

# Supplemental Figure 12. Probability sensitivity analysis impacts on economic costs and QALYs per treatment arm, Belarus

Notes: QALYs= Quality-adjusted life years. mSTR= modified shorter all-oral treatment regimen. USD= United States dollars.

# Supplemental Figure 13. Percentage of simulations per incremental ICER and QALY scenarios, Belarus

Notes: QALYs= Quality-adjusted life years. mSTR= modified shorter all-oral treatment regimen.

# Supplemental Figure 14. Probability sensitivity analysis impacts on economic costs and QALYs per treatment arm, Georgia

Notes: QALYs= Quality-adjusted life years. mSTR= modified shorter all-oral treatment regimen. USD= United States dollars.

# Supplemental Figure 15. Percentage of simulations per incremental ICER and QALY scenarios, Georgia

Notes: QALYs= Quality-adjusted life years. mSTR= modified shorter all-oral treatment regimen.

# Supplemental Figure 16. Costs-effectiveness acceptability curves (CEAC), by country

Notes: WTP= Willingness-to-pay. ICER= Incremental cost-effectiveness ratio. Dotted vertical lines represent the maximum country-specific acceptability threshold for the mSTR treatment, compared to SOC, based on Woods *et al 2016*^2^. See Table A4 for more information on acceptability thresholds. The 5% band (shaded area around the CEAC) was selected based on the approximate range of variation observed across repeated probabilistic sensitivity analyses, serving as a pragmatic visual proxy for uncertainty in the absence of full simulation-based confidence intervals. mSTR was cost-effective, compared to SOC, in 74%, 75%, 88% and 90% of PSA simulations in Belarus, Georgia, Moldova, and Kazakhstan, respectively.

# Supplemental Figure 17. Incremental Net Monetary Benefit (iNMB) across WTP thresholds

Notes: WTP= Willingness-to-pay. ICER= Incremental cost-effectiveness ratio. USD= United States dollars. QALY= Quality adjusted life year. iNMB= Incremental net monetary benefit. The blue shaded area indicates the interquartile range (are between percentile 25^th^ and 75^th^ of the iNMB values). Mean iNMB and shaded area were computed from all 1,000 Monte Carlo data points per country for simulations of incremental costs and QALYs.

# Supplemental Figure 18. Expected Value of Perfect Information (EVPI) across different WTP thresholds, by country

Notes: WTP= Willingness-to-pay. ICER= Incremental cost-effectiveness ratio. USD= United States dollars. QALY= Quality adjusted life year. iNMB= Incremental net monetary benefit.

# Supplemental Figure 19. Costs and cost-savings at scale

Notes: RR= Rifampicin resistance, MDR= Multidrug-resistance, TB= Tuberculosis, SOC= Standard of care, mSTR= Modified shorter all-oral treatment regime. Incidence of MDR/RR TB and percentage of individuals under mSTR obtained from Table 1. National TB budget extracted from WHO’s website. Costs and cost-savings per patient were obtained from Table 2.

# Supplemental Figure 20. Impact of mSTR scale-up on MDR/RR-TB treatment expenditure, by country

Notes: *Current mSTR coverage is based on country level estimates (Belarus=34%, Georgia=57%, Kazakhstan 6%, Moldova=19%). An 80% upper limit for mSTR scale-up was assumed to reflect eligibility criteria, with the remaining 20% of notifications assumed to receive SOC. Similarly, 0% mSTR scenario assumes 100% coverage of SOC. Total TB budgets in each country were sourced from WHO estimates^31^. See Table A16 for the full budget scenario analysis results. MDR= Multidrug resistance. RR-TB= Rifampicin-resistant tuberculosis.

# References

1.	Pontali E, Raviglione MC, Migliori GB. Regimens to treat multidrug-resistant tuberculosis: past, present and future perspectives. *European Respiratory Review* 2019; **28**(152).

2.	Woods B, Revill P, Sculpher M, Claxton K. Country-level cost-effectiveness thresholds: initial estimates and the need for further research. *Value in Health* 2016; **19**(8): 929-35.

3.	Husereau D, Drummond M, Augustovski F, et al. Consolidated Health Economic Evaluation Reporting Standards 2022 (CHEERS 2022) statement: updated reporting guidance for health economic evaluations. *International journal of technology assessment in health care* 2022; **38**(1): e13.
